# Correlation Between Enhanced Magnetic Resonance Exam and Pure-Tone Audiometry Tests of Patients With Meniere’s Disease

**DOI:** 10.7759/cureus.97275

**Published:** 2025-11-19

**Authors:** Henrique F Pauna, Jéssica Echeverria, Eduarda B Loh, Alexandre C Gasperin

**Affiliations:** 1 Otolaryngology, Hospital Universitário Cajuru, Curitiba, BRA; 2 Otolaryngology - Head and Neck Surgery, Pontifícia Universidade Católica do Paraná, Curitiba, BRA

**Keywords:** dizziness, endolymphatic hydrops, hearing loss, magnetic resonance, meniere's disease, tinnitus

## Abstract

Introduction: Meniere's disease (MD) is a disease affecting the inner ear that is attributed to endolymphatic hydrops. Several electrophysiological tests and imaging techniques have been developed to improve the diagnosis of endolymphatic hydrops.

Objective: This study aimed to correlate inner ear magnetic resonance findings after intravenous gadolinium injection with the results of pure-tone audiometry tests for the diagnosis of MD.

Methods: Patients meeting the criteria for a diagnosis of MD were retrospectively included from January 2016 to May 2021 at Hospital IPO (Instituto Paranaense de Otorrinolaringologia)/Paraná Institute of Otolaryngology, a private hospital in Paraná, Brazil. The endolymphatic hydrops was graded in four degrees on the gadolinium-enhanced magnetic resonance of the inner ear based on the inversion of the saccule to utricle ratio, as indicated by Reissner’s membrane distension. Correlations were made with pure-tone audiogram, using Spearman’s coefficient, Fisher’s exact test, and non-parametric tests.

Results: Fifty-three patients were evaluated (totaling 106 ears). The most common symptoms reported by patients were tinnitus (86.8%), dizziness (83%), hearing loss (81.1%), and ear fullness (64.2%). Vestibular endolymphatic hydrops was observed in 77.4% of the affected ears, while cochlear endolymphatic hydrops was present in 50.9%. Differences in hearing thresholds were found between affected ears and controls at all frequencies analyzed, with statistical significance.

Conclusions: Our study demonstrated that endolymphatic hydrops can be identified through magnetic resonance contrast-enhanced with gadolinium, showing that the same patient may have different degrees of dilatation of the membranous labyrinth between their two ears. We observed significant differences in pure-tone thresholds in audiometry tests according to the progression of the degree of endolymphatic hydrops.

## Introduction

Meniere's disease (MD) is a poorly understood inner ear disorder characterized by spontaneous attacks of vertigo, fluctuating hearing loss, tinnitus, and aural fullness. Its pathological hallmark is endolymphatic hydrops (EH) [1]. 

The clinical diagnostic criteria for MD, jointly formulated by the American Academy of Otolaryngology-Head and Neck Surgery (AAO-HNS) and the Classification Committee of the Bárány Society, include two categories: "definite" and "probable" MD. The clinical features of MD are easily confused with vestibular migraine, psychogenic vertigo, and benign recurrent vertigo; thus, the differential diagnosis of MD remains very challenging. In the 1995 AAO-HNS criteria (updated in 2015), the category of "definite MD" was based on post-mortem histological evidence of EH. With this new classification, defined forms of MD require the observation of two or more episodes of vertigo associated with low to medium frequency sensorineural hearing loss (documented by audiometry) on at least one occasion and fluctuating auditory symptoms (hearing loss, tinnitus, and aural fullness) in the affected ear [2,3]. “Probable MD” is defined by two or more episodes of vertigo with fluctuating auditory symptoms in the affected ear [3].

In the mid-2000s, a new method for visualization of EH was described, using 3 Tesla nuclear magnetic resonance (MRI) examination with linear gadolinium (Gd) enhancement [4-6]. Intratympanic (IT) or intravenous (IV) injections of Gd for visualization of EH are described. The IV method is less invasive and is not influenced by factors affecting the round window membrane, and the contrast is distributed more evenly in the inner ear compartment as compared with the IT method. In addition, the IV method allows simultaneous visualization of the bilateral labyrinth, allowing the MRI to verify the permeability of the blood-labyrinth barrier in both ears [7].

MRI studies using the three-step classification system showed a percentage of EH that ranged from 47% to about 90% in the symptomatic ear and a high rate of EH in asymptomatic ears [3].

However, a recent case-control study found that the semiquantitative system used to classify EH did not allow discriminating symptomatic individuals from healthy volunteers [8]. The limits of the semiquantitative approach depended on the time of inversion of the MRI sequence that could modify the ratio between endolymph and perilymphatic fluid, and the criteria for cochlear hydrops were based on slight dilatation of the endolymphatic duct, which is also found in healthy temporal bones.

Currently, the diagnosis of MD is based on complementary assessments. Combining the volumetric reconstruction method in inner ear MRI, the endolymphatic space and EH can be evaluated objectively, quantitatively, and precisely [6].

EH is considered the most common finding of MD, according to studies with human temporal bones and imaging. However, the assumption that EH is the direct cause of symptoms has been questioned because hydrops may be a coincident finding in asymptomatic patients. Thus, it is questioned whether there is a correlation between the MRI findings of the inner ear after injection of gadolinium contrast with the electrophysiological tests used to diagnose MD. This study aims to correlate the changes in the membranous labyrinth evaluated by late magnetic resonance examination of the inner ear after intravenous gadolinium injection, with pure-tone audiometry tests in patients with a suspected diagnosis of MD.

## Materials and methods

A retrospective study was carried out, analyzing the medical records of patients seen at the neurotology outpatient clinic of Hospital IPO (Instituto Paranaense de Otorrinolaringologia)/Paraná Institute of Otolaryngology, a private hospital in Paraná, Brazil, between January 2016 and May 2021, who had been diagnosed with MD. The research protocol was authorized by the Research Ethics Committee of the hospital (approval no. 4,216,682). Patients with dizziness undergo a systematic routine of hearing and vestibular evaluation in our institution, which includes pure-tone audiometry. We included adult patients (over 18 years old) with complaints suggestive of the diagnosis of MD. We excluded patients with bilateral MD, surgical ear diseases (such as chronic otitis media, chronic cholesteatomatous otitis media), patients with previous ear surgeries, a history of metastatic diseases, and a history of use of ototoxic or vestibulotoxic medications. Patients with bilateral MD were excluded because the study design required comparison between the affected ear and the contralateral ear. Patients who did not fully answer the questionnaires in this study were also excluded.

Radiological evaluation

The selected subjects were referred for a nuclear magnetic resonance exam in a private clinic, following an intravenous gadolinium contrast injection protocol, four hours before the imaging exam. The image acquisition sequence was performed according to the protocol proposed by Bernaerts et al. (2019) [9].

The degree of EH in the vestibule and cochlea was assessed by visual comparison of the relative areas of the unenhanced endolymphatic space versus the contrast-enhanced perilymph space in the axial plane, separately for the cochlea and the vestibule. The degree of cochlear hydrops was classified as none (grade 0), grade I, grade II, or grade III, according to the criteria previously described by Baráth et al. (2014) [10].

Visual assessment of the saccule/utricle ratio was performed on axial images at the lowest level of the vestibule, as, according to histological studies, the saccule occupies the lower, medial, and anterior part of the vestibule (Lane et al., 2008) [11]. The degree of perilymphatic enhancement was also evaluated semi-quantitatively in all ears, visually comparing the degree of involvement of one ear in relation to the contralateral ear.

**Figure 1:**
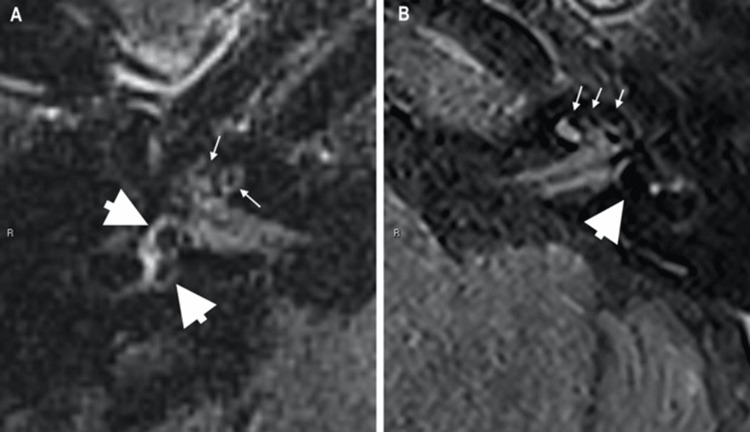
Radiographic evaluation of membranous labyrinth distension. A: axial view of the right ear MRI, classified as grade I. B: axial view of the left ear MRI, classified as grade III. Smaller arrow: cochlear distension; larger arrow: vestibular distension.

We classified vestibular hydrops into four degrees (0 to III). Grade 0 is equivalent to the absence of distension of the membranous labyrinth. Grade I when the smallest of the two vestibular saccules is equal to or larger than the utricle, but not yet confluent (with the utricle). For grade III, the assessment of vestibular perilymphatic enhancement was considered when there was no visible perilymphatic space to assess. Grade II, on the other hand, defines intermediate changes between the findings of grades I and III (Figure 1).

Audiovestibular evaluation

The standard pure-tone audiometry was obtained at frequencies of 250, 500, 1000, 2000, 3000, 4000, 6000, and 8000 Hz. The mean of the pure tone was calculated using the frequencies of 500, 1000, 2000, and 4000 Hz. Sensorineural hearing loss ranged from mild (26 to 40 dB), moderate (41 to 70 dB), severe (71 to 90 dB), and profound (> 91 dB). Patients with bilateral auditory symptoms (i.e., hearing loss, tinnitus, and ear fullness) were considered to have bilaterally affected ears.

Statistical analysis

The results of quantitative variables were described as mean, standard deviation, median, minimum, and maximum. For categorical variables, frequency and percentage were presented. The evaluation of the correlation between two quantitative variables was carried out by estimating the Spearman coefficient. To analyze the association between two categorical variables, Fisher's exact test was used. The comparison between two groups defined by classifications of categorical variables, concerning hearing threshold, was made using the non-parametric Mann-Whitney test. For the comparison between affected and control ears, concerning quantitative variables, the non-parametric Wilcoxon test was used. Categorical variables were analyzed using the binomial test. The condition of normality of continuous quantitative variables was assessed using the Kolmogorov-Smirnov test. Values ​​of p < 0.05 indicated statistical significance. Data were analyzed using the computer program Stata/SE release 14.1 (StataCorp LLC, StataCorp., College Station, TX, 2015).

## Results

A total of 53 patients, 30 female (56.6%) and 23 male (43.4%), were included, totaling 106 ears evaluated (the ear contralateral to the symptoms was considered the control ear). The mean age at onset of symptoms was 42.5 ± 13.4 years. The median age at diagnosis was 46.6 years (ranging from 25.2 to 78.7 years). The mean age for the MRI examination was 47.7 ± 12.6 years. Finally, the time elapsed between the onset of symptoms and the diagnostic conclusion was 4.4 ± 5.2 years.

**Figure 2:**
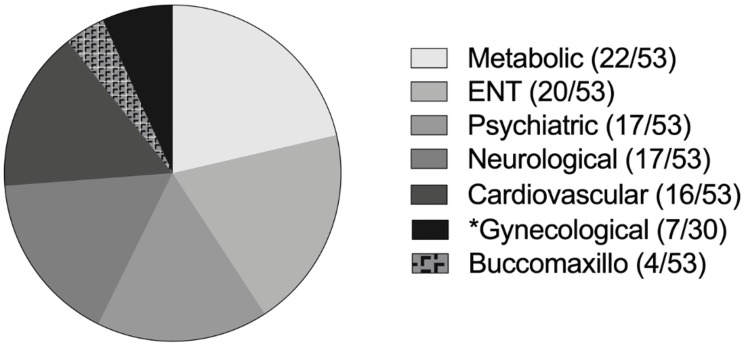
Number of patients by a group of pathologies. *Only in the female group

The presence of other associated comorbidities was recorded, as well as the number of medications that the patients responded to taking during routine consultations. Tables 1-2 illustrate the distribution of pathology groups, as well as the number of patients in each group, included in this study (Tables 1-2, Figure 2). What was observed is that the patients included in this study use, on average, 5.2 ± 3.5 medications. We did not find significant differences between genders regarding the presence of associated pathologies (p > 0.05; Fisher's exact test).

**Table 1:** Associated pathologies.

Variable	Total	Response	n	%
Number of associated pathologies	53	0	4	7.5
		1	17	32.1
		2	14	26.4
		3 or more	18	33.9
Number of associated pathologies (grouped)	53	0 or 1	21	39.6
		More than 1	32	60.4

**Table 2:** Number of medications according to associated pathologies.

Variable	Total	Number of medications	n	%
Psychiatric	53	0	32	60.4
		1	9	17.0
		2	4	7.5
		3 or more	8	15.1
Metabolic	53	0	32	60.4
		1	14	26.4
		2	3	5.7
		3 or more	4	7.6
Neurological	53	0	39	73.6
		1	10	18.9
		2	3	5.7
		3 or more	1	1.9
Otorhinolaryngological	53	0	5	9.4
		1	10	18.9
		2	15	28.3
		3 or more	23	43.4
Cardiovascular	53	0	36	67.9
		1	9	17.0
		2	5	9.4
		3 or more	3	5.7
Odontological	53	0	48	90.6
		1	5	9.4
Gynecological	30	0	28	93.3
(restricted to women)		1	2	6.7
Number of medications	53	Up to 5	32	60.4
		More than 5	21	39.6

Among the complaints associated with the classic tetrad attributed to clinical suspicion of MD, the symptom that was most frequently reported by the patients was tinnitus. Among the patients evaluated, 46 presented with tinnitus (86.8%), 44 (83%) reported dizziness, 43 (81.1%) hearing loss, and 34 (64.2%) aural fullness. We observed that 21 patients (41.5%) had three of these four symptoms, followed by 20 patients (37.7%) with all tetrad symptoms, nine patients (18.9%) with only two of the symptoms, and only one patient had a single symptom of a classical tetrad.

When we analyzed the association between associated comorbidities and the number of symptoms of the classic tetrad, we did not obtain a positive association (p > 0.05; Fisher's exact test).

**Figure 3:**
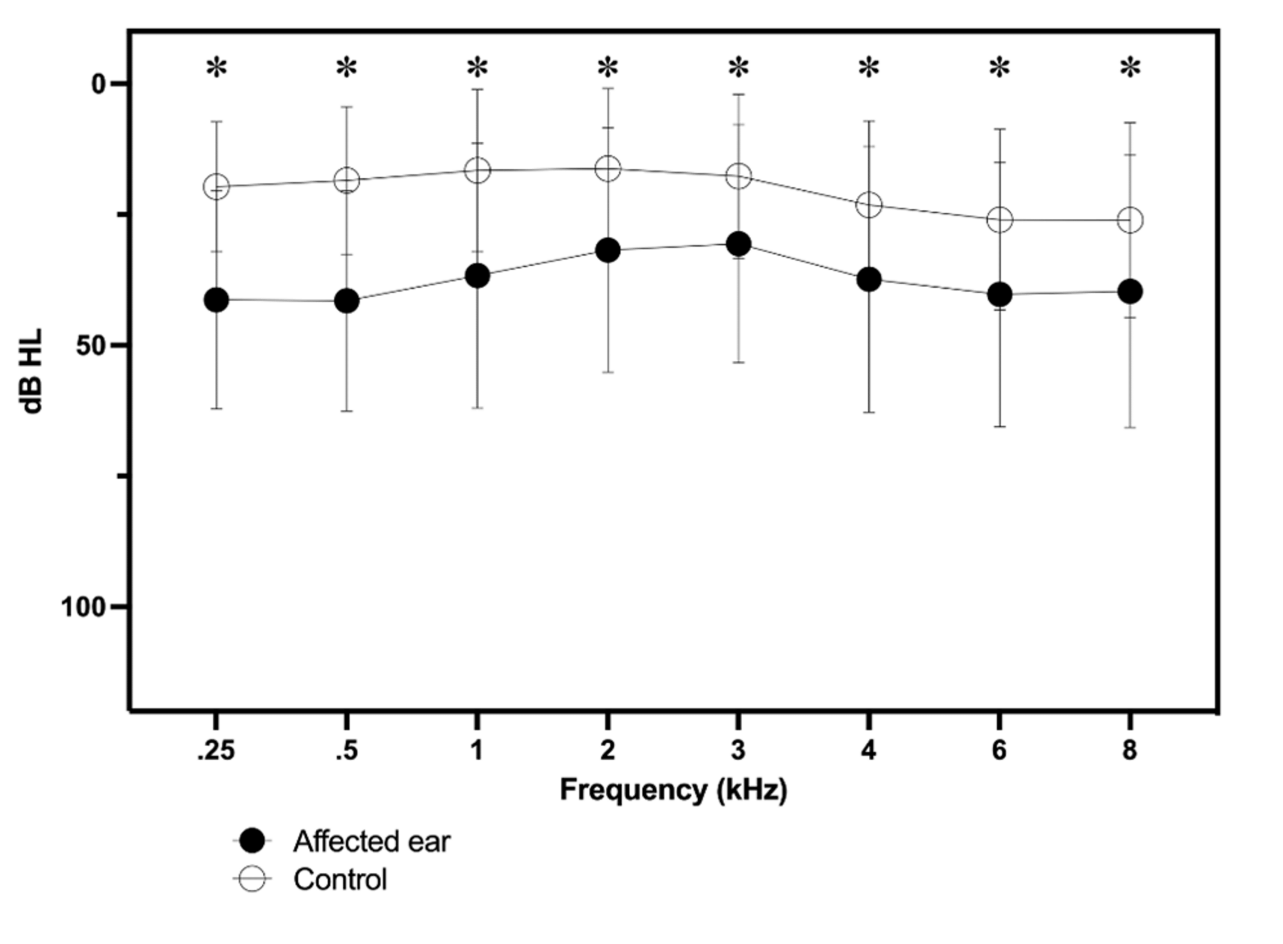
Tonal thresholds between the affected ear and control ear. *p < 0.05, non-parametric Wilcoxon test

Figure 3 shows the means of pure-tone thresholds, between 250 and 8000 Hz, between the affected and control ears of the patients included in the study. We observed a significant difference for all frequencies analyzed when comparing affected and control ears (p < 0.001). For each frequency and each ear, the correlation between the hearing threshold and the number of medications was tested (Table 3). When grouped into two different groups (those patients who use up to five medications or those who use more than five medications), we found significant differences in the means of pure-tone thresholds according to the frequency ranges evaluated. Those patients who used a greater number of medications had higher pure-tone thresholds (Table 4).

**Table 3:** Correlation between hearing thresholds and the number of medications (n = 53).

Variable	Spearman	p
Affected ear		
Number of medications x 250	0.2	0.146
Number of medications x 500	0.2	0.147
Number of medications x 1000	0.29	0.038
Number of medications x 2000	0.40	0.003
Number of medications x 3000	0.51	<0.001
Number of medications x 4000	0.47	<0.001
Number of medications x 6000	0.40	0.003
Number of medications x 8000	0.39	0.004
Control ear		
Number of medications x 250	0.10	0.464
Number of medications x 500	0.15	0.298
Number of medications x 1000	0.16	0.255
Number of medications x 2000	0.39	0.004
Number of medications x 3000	0.38	0.005
Number of medications x 4000	0.33	0.017
Number of medications x 6000	0.42	0.002
Number of medications x 8000	033	0.016

**Table 4:** Comparison of pure-tone threshold means between patients who use up to five medications and with those who use more than five medications, associating the affected and control ear. *Mann-Whitney non-parametric test, p < 0.05

Frequency (Hz)	Medications	n	Avarage ± SD (dB)	Avarage (min–max) (dB)	p*
Affected ear					
250	≤ 5	32	39.5 ± 19	40 (0–80)	0.546
	> 5	21	44 ± 23.6	40 (5–85)	
500	≤ 5	32	40.2 ± 18.6	40 (0–65)	0.752
	> 5	21	43.6 ± 24.9	40 (10–95)	
1000	≤ 5	32	32.2 ± 19.9	30 (0–65)	0.195
	> 5	21	43.6 ± 31.2	40 (5–110)	
2000	≤ 5	32	24.4 ± 18.4	20 (0–65)	0.009
	> 5	21	43.1 ± 26.1	40 (5–90)	
3000	≤ 5	32	22 ± 16.7	15 (0–70)	0.001
	> 5	21	45.5 ± 25.1	45 (5–100)	
4000	≤ 5	32	29.5 ± 20.9	25 (0–90)	0.007
	> 5	21	43.6 ± 23.3	45 (0–90)	
6000	≤ 5	32	33.6 ± 21.5	35 (0–85)	0.022
	> 5	21	50.5 ± 27.7	50 (5–120)	
8000	≤ 5	32	33.1 ± 23.3	30 (0–100)	0.037
	> 5	21	49.8 ± 27.7	55 (10–110)	
Control Ear					
250	≤ 5	32	18.8 ± 9.9	15 (0–50)	0.510
	> 5	21	21.2 ± 15.6	20 (0–80)	
500	≤ 5	32	16.7 ± 10.4	15 (0–50)	0.432
	> 5	21	21.2 ± 18.3	15 (5–90)	
1000	≤ 5	32	13.1 ± 8.2	12.5 (0–35)	0.222
	> 5	21	21.9 ± 21.6	15 (5–85)	
2000	≤ 5	32	11.7 ± 8.6	10 (0–40)	0.019
	> 5	21	23.1 ± 20.4	15 (0–85)	
3000	≤ 5	32	12.7 ± 9.0	10 (0–30)	0.015
	> 5	21	25.5 ± 20.2	15 (5–80)	
4000	≤ 5	32	18.8 ± 10.8	17.5 (0–40)	0.048
	> 5	21	30 ± 20.2	25 (5–85)	
6000	≤ 5	32	21.4 ± 12.8	20 (0–50)	0.032
	> 5	21	33.1 ± 20.9	30 (0–90)	
8000	≤ 5	32	21.4 ± 13.5	20 (0–55)	0.055
	> 5	21	33.3 ± 22.9	25 (5–95)	

The MRI examination with intravenous gadolinium contrast was performed and graded by an experienced radiologist. Table 5 shows the results of the radiological evaluation of the MRI examinations of both ears of the patients included in the study.

**Table 5:** Results of the assessment of the degree of hydrops according to the assessed ear. *Binomial test, p < 0.05

Variable	Total	Level	Affected		Control				p*
			n	%	n	%			
Vestibular hydrops	53	0	12	22.6	29	54.7		-	
		1	10	18.9	9	17.0			
		2	20	37.7	14	26.4			
		3	11	20.8	1	1.9			
Vestibular hydrops (grouped)	53	0 or 1	22	41.5	38	71.7		<0.001	
		2 or 3	31	58.5	15	28.3			
Cochlear hydrops	53	0	26	49.1	42	79.2		-	
		1	15	28.3	11	20.8			
		2	12	22.6	-	-			
		3	-	-	-	-			
Cochlear hydrops (grouped)	53	0 or 1	41	77.4	53	100		<0.001	
		2 or 3	12	22.6	-	-			

**Figure 4:**
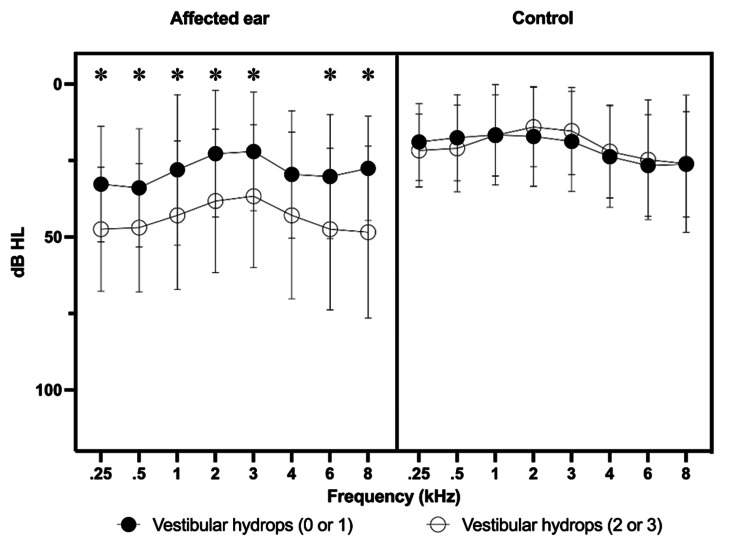
Correlation between pure-tone thresholds at the different frequencies evaluated and degree of vestibular hydrops in the affected ears and those in the control group. *Mann-Whitney non-parametric test, p < 0.05

**Figure 5:**
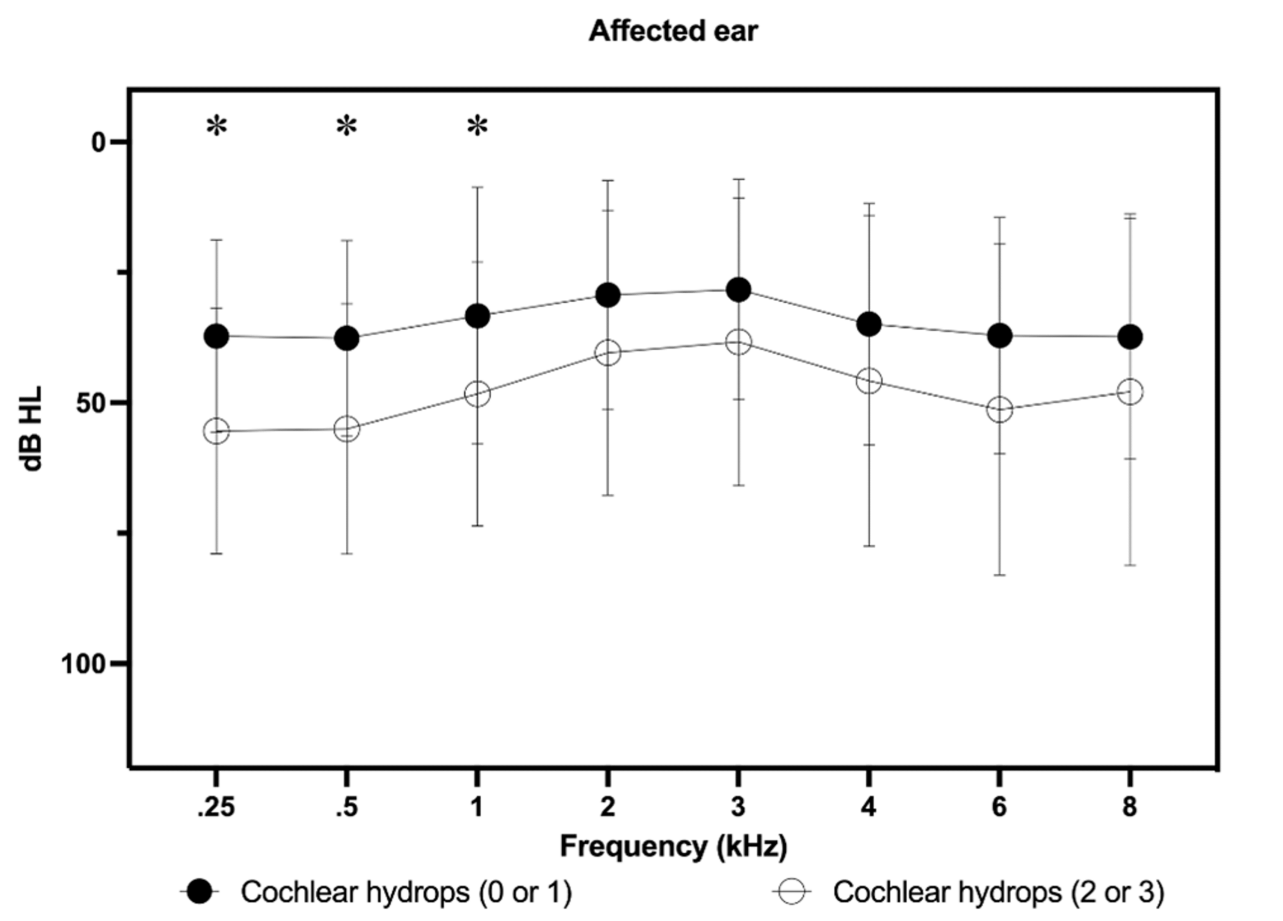
Correlation between pure-tone thresholds in the different frequencies evaluated and degree of cochlear hydrops in the affected ears. No analysis of auditory thresholds and comparisons with the control ear was performed, as there were no patients in the control group classified as having grade 2 or 3 cochlear hydrops. *Mann-Whitney non-parametric test, p < 0.05

The present study correlated the results of the degrees of hydrops (vestibular and cochlear) in each ear (affected and control), in all audiometric frequencies separately. This search did not find a statistically significant correlation (Figures 4, 5).

We evaluated the possible association between pure-tone thresholds at each of the frequencies studied with the symptoms presented by the patients. When analyzing pure-tone thresholds with complaints, we observed a correlation between hearing loss and pure-tone thresholds at 1000 Hz in the affected ear (p = 0.043; Mann-Whitney test), complaints of fullness, and pure-tone thresholds at 250 Hz in the affected ear (p = 0.018; Mann-Whitney test), complaint of fullness and pure-tone thresholds at 6000 Hz in the control ear (p = 0.024; Mann-Whitney test), and complaint of tinnitus and pure-tone thresholds in 1000 Hz in the affected ear (p = 0.04; Mann-Whitney). When we consider the complaint of dizziness, we did not obtain a statistical correlation in the affected or control ear. When considering the tinnitus complaint, there was a correlation of pure-tone thresholds only in the frequency of 1000 Hz of the affected ear (p = 0.040; non-parametric Mann-Whitney test). When considering the complaint of hearing loss, we found a significant association at 1000 Hz in the affected ear (p = 0.043). Regarding fullness, we found a significant association at 250 Hz in the affected ear (p = 0.018) and 6000 Hz in the control ear (p = 0.024). 

For each symptom reported by the patient (dizziness, tinnitus, hearing loss, or fullness), the correlation with the presence of hydrops (vestibular or cochlear) was tested. We did not obtain a correlation between these analyzed variables (p > 0.05; Fisher's exact test).

## Discussion

MD is an inner ear disorder of multifactorial origin characterized by distension of the endolymphatic membranous labyrinth, detected through histological studies of temporal bones after an autopsy, that is, limiting the monitoring of the disease-related pathological process [12]. By allowing visualization of the boundary between the endolymphatic and perilymphatic spaces, MRI images after gadolinium contrast injection facilitated the detection of hydrops in living patients [12]. In the present study, we performed a retrospective analysis of MRI exams contrasted with intravenous gadolinium to visualize the presence and degree of endolymphatic hydrops in the vestibule and cochlea, correlating the different degrees of hydrops with the auditory thresholds and with the clinical variables of the population included.

In the present study, 12 (22,64%) of the 53 exams evaluated did not show dilation of the membranous labyrinth in the affected ear (side of the patient's main complaint). Asymptomatic vestibular endolymphatic hydrops in the contralateral ear was observed in 24 patients. Asymptomatic endolymphatic hydrops occurred more in the vestibular membranous labyrinth (24 ears) than in the cochlear (11 ears), unlike the study by Li et al. [12]. Previous studies suggest that bilateral hydrops does not necessarily correlate with clinical signs of bilateral MD. Asymptomatic hydrops has been considered more of a finding than a cause of MD [13], although the dilatation of the membranous labyrinth, observed in histopathological studies of temporal bones in 100% of patients with a history of MD, supports the hypothesis that endolymphatic hydrops causes MD [14]. Attyé et al. [8] evaluated the degree of utricle distension as a reference for saccular hydrops, using the inversion of the saccule/utricle ratio, and demonstrated that this inversion is a reliable qualitative marker of endolymphatic hydrops, with specificity and sensitivity of 100% and 50%, respectively. Our study observed significant differences in pure-tone thresholds in audiometry tests according to the progression of the degree of EH, and vestibular hydrops was more common (31 patients (58.5%) of cases classified as grade 2 or 3), consistent with the study by Yoshida et al. (2018) [15] who considered vestibular hydrops to be a more specific prognostic factor than cochlear hydrops for the definitive diagnosis of MD.

The results observed in this study support the theory that there is increased permeability of the blood-labyrinthine barrier. The increase in perilymphatic enhancement in the affected ear of patients with MD has already been observed in previous radiological studies [9]. It is also suggested that when the endolymphatic pressure exceeds the saccular compliance, there is a failure in the regulation of endolymph in the inner ear, leading to lesions of the stereocilia of the inner and outer hair cells, early changes in spiral ligament fibrocytes, or changes in cell morphology (such as in Reissner's membrane distension) [16]. An otopathological study by Okuno and Sando (1987) demonstrated that hydrops is more frequent in the inferior pars (cochlea and saccule) than in the superior pars (utricle and semicircular canals). Due to the characteristic of the distribution of vestibular dark cells (ampulla of the semicircular canals, utricle, and crus commune) and the morphology and functioning of the Bast valve, it is to be assumed that endolymph is produced by cochlear dark cells located in the stria vascularis, emphasizing the importance of this structure (a component of the blood-labyrinth barrier) in the pathogenesis of MD [12,17,18].

An additional finding of our study was the association between the number of medications used by the patients and the deterioration of hearing thresholds, both in the affected and control ears. Patients taking more than five medications showed significantly higher pure-tone thresholds, particularly at mid and high frequencies. This result may reflect the influence of systemic comorbidities and polypharmacy on cochlear metabolism and microcirculation. Previous studies have suggested that certain medications, such as antihypertensives, diuretics, and psychotropic drugs, can alter inner ear homeostasis and affect auditory function, especially in patients with preexisting endolymphatic hydrops. Although our study did not investigate the specific pharmacological mechanisms involved, the observed correlation highlights the importance of considering polypharmacy and systemic health factors when interpreting audiometric findings in patients with MD.

The present study has some limitations that should be acknowledged. The relatively small sample size restricts the statistical power and limits the external validity of the findings, and the use of the contralateral healthy ear as a control group may not constitute an ideal comparison. Furthermore, some imaging examinations were conducted outside episodes of vertigo or hypoacusis, which may have influenced the accuracy and representativeness of the results. Another limitation of this study is that the correlation between the duration of symptoms and the imaging or audiometric findings was not evaluated, which could provide further insights into disease progression.

## Conclusions

The results demonstrated that endolymphatic hydrops can be reliably identified through delayed post-contrast MRI and that progressive degrees of hydrops were associated with significant differences in hearing thresholds. Although the correlations between imaging severity and clinical measures were modest, these findings reinforce the complementary role of MRI in conjunction with audiometric evaluation for a more comprehensive understanding of MD. The limitations related to the retrospective design and the use of the contralateral ear as a control should be considered when interpreting the results. Future prospective studies with larger samples are encouraged to further clarify the relationship between disease progression, imaging findings, and auditory function.
